# Gene Expression Analysis of the Bone Marrow Microenvironment Reveals Distinct Immunotypes in Smoldering Multiple Myeloma Associated to Progression to Symptomatic Disease

**DOI:** 10.3389/fimmu.2021.792609

**Published:** 2021-11-22

**Authors:** Ignacio Isola, Fara Brasó-Maristany, David F. Moreno, Mari-Pau Mena, Aina Oliver-Calders, Laia Paré, Luis Gerardo Rodríguez-Lobato, Beatriz Martin-Antonio, María Teresa Cibeira, Joan Bladé, Laura Rosiñol, Aleix Prat, Ester Lozano, Carlos Fernández de Larrea

**Affiliations:** ^1^ Department of Hematology, Hospital Clínic de Barcelona, Institut d’Investigacions Biomèdiques August Pi i Sunyer (IDIBAPS), University of Barcelona, Barcelona, Spain; ^2^ Department of Medical Oncology, Hospital Clinic de Barcelona, Institut d’Investigacions Biomèdiques August Pi i Sunyer (IDIBAPS), Barcelona, Spain; ^3^ Department of Cell Biology, Physiology and Immunology, Faculty of Biology, University of Barcelona, and Institute of Biomedicine of the University of Barcelona (IBUB), Barcelona, Spain; ^4^ Experimental and Clinical Hematology Program (PHEC), Josep Carreras Leukaemia Research Institute, Barcelona, Spain

**Keywords:** smoldering multiple myeloma, immunotherapy, immune checkpoints, TIGIT, pronostic factors, bone marrow microenvironment

## Abstract

**Background:**

We previously reported algorithms based on clinical parameters and plasma cell characteristics to identify patients with smoldering multiple myeloma (SMM) with higher risk of progressing who could benefit from early treatment. In this work, we analyzed differences in the immune bone marrow (BM) microenvironment in SMM to better understand the role of immune surveillance in disease progression and to identify immune biomarkers associated to higher risk of progression.

**Methods:**

Gene expression analysis of BM cells from 28 patients with SMM, 22 patients with monoclonal gammopathy of undetermined significance (MGUS) and 22 patients with symptomatic MM was performed by using Nanostring Technology.

**Results:**

BM cells in SMM compared to both MGUS and symptomatic MM showed upregulation of genes encoding for key molecules in cytotoxicity. However, some of these cytotoxic molecules positively correlated with inhibitory immune checkpoints, which may impair the effector function of BM cytotoxic cells. Analysis of 28 patients with SMM revealed 4 distinct clusters based on immune composition and activation markers. Patients in cluster 2 showed a significant increase in expression of cytotoxic molecules but also inhibitory immune checkpoints compared to cluster 3, suggesting the presence of cytotoxic cells with an exhausted phenotype. Accordingly, patients in cluster 3 had a significantly longer progression free survival. Finally, individual gene expression analysis showed that higher expression of TNF superfamily members (TNF, TNFAIP3, TNFRSF14) was associated with shorter progression free survival.

**Conclusions:**

Our results suggest that exhausted cytotoxic cells are associated to high-risk patients with SMM. Biomarkers overexpressed in patients with this immune gene profile in combination with clinical parameters and PC characterization may be useful to identify SMM patients with higher risk of progression.

## Introduction

Smoldering multiple myeloma (SMM) is a pre-malignant condition that precedes symptomatic MM and is defined by a serum monoclonal immunoglobulin (M-protein) of ≥3 g/dL and/or an urinary monoclonal protein ≥500 mg per 24 h, and/or 10–60% clonal bone marrow plasma cells (BMPC), in the absence of end-organ damage (1, 2). Since only a fraction of SMM patients will progress to active MM, the initiation of an early anti-myeloma treatment is a subject of intense discussion. Based on the revised International Myeloma Working Group (IMWG) criteria, asymptomatic patients with ultra-high risk SMM are currently considered to have active MM and treatment is recommended (2, 3). We previously defined two subsets of patients with SMM: 1- the ‘evolving’ variant of SMM, characterized by a progressive increase in the M-protein size until symptomatic myeloma develops and a shorter time to progression and 2- the non-evolving pattern, with a long-lasting stable M-protein and a longer time to progression (4). The actuarial transformation rates at 10 years of follow-up were 55 and 10% in patients with ‘evolving’ and ‘non-evolving’ pattern, respectively (1, 4). In line with these results, we recently evaluated progression risk factors in 206 patients with SMM, demonstrating that median time from recognition of evolving type to progression into symptomatic MM was 1.1 years and progression rate at 3 years was 71% (5). Therefore, confirmation of an evolving behavior drastically worsened the prognostic estimation made at diagnosis for every covariate predictive of progression (serum M-protein size, BMPC infiltration, immunoparesis and Mayo Clinic risk score) (5). Accordingly, the revised IMWG risk stratification model for SMM (“2/20/20”) identified three independent factors predicting progression risk at 2 years: serum M-protein >2 g/dL, involved to uninvolved free light-chain (FLC) ratio >20, and BMPC infiltration >20% (6).

One of the main challenges of assessing progression risk is to take into account the BM heterogeneity in molecular and cellular patterns that leads to the different clinical behavior of patients included under the designation of SMM (7). Given the relevance of microenvironment for malignant PC survival and the absence of a clear molecular “second hit” between SMM and symptomatic MM (8), it is crucial to find new immune biomarkers associated to the risk of progression to symptomatic MM that allow us to evaluate the need for an early intervention (9, 10). In this study we aim to investigate whether molecular and cellular mechanisms in the BM immune microenvironment may explain the heterogeneity observed in the clinic. Our goal is to better understand the composition and functional levels of BM cells surrounding malignant PC and correlate this molecular data with clinical behavior to identify key molecules and cell types associated to progression from SMM to active myeloma.

## Materials And Methods

### Patient Cohorts

BM aspiration samples were collected from 28 patients with SMM at diagnosis (patient characteristics are summarized in **Table 1**). In addition, for comparison purposes we also studied BM samples from 22 patients with MGUS and from 22 patients with symptomatic MM (12 refractory/relapsed MM patients and 10 newly diagnosed untreated patients, 5 of them corresponding to patients also analyzed at the SMM stage who later progressed, allowing a paired comparison).

**Table 1:** Patient characteristics.

	MGUS	SMM	MM
Number of patients	22	28	22
Age, median years (range)	70 (40–88)	69 (38–84)	68 (49–80)
Gender, male/female	12/10	11/17	11/11
Isotype (%)			
• IgG	15 (68)	15 (54)	12 (55)
• IgA	7 (32)	11 (39)	10 (45)
• Biclonal		1 (3,5)	
• Light chain		1 (3,5)	
Serum M-protein g/L*	15.3 (13.1-20.6)	20.2 (13.4-32.1)	25.8 (11.6-40.8)
Serum FLCr*	2.2 (0.1-8.8)	1.5 (0.7-11.3)	33.4 (2.7-423)
BMPC (%)*	5.5 (3–8)	19 (12.5 -24.7)	34 (15.5-47)
Abnormal BMPC (%)*^Ɨ^	71.5 (33.2-85.7)	98 (96.7-100)	100 (99–100)
ISS stage (%)	—	—	
I			5 (26)
II			7 (37)
III			7 (37)
Risk Stage (%)^‡^			—
Low	2 (9)	11 (41)	
Intermediate	17 (77)	8 (29.5)	
High	3 (14)	8 (29.5)	

SMM, smoldering multiple myeloma; MGUS, monoclonal gammopathy of undetermined significance; MM, symptomatic multiple myeloma; FLCr, serum free light chain ratio (kappa/lambda); BMPC, bone marrow plasma cell count.

ISS, International staging system for multiple myeloma.

*Measurements are median (interquartile range).

^Ɨ^Percentage of bone marrow plasma cells with abnormal phenotype by flow cytometry.

^‡^The International Myeloma Working Group (IMWG) SMM revised risk model includes serum M-protein >2 g/dL, involved to uninvolved free light-chain ratio >20 and bone marrow plasma cell infiltration >20%. The Mayo Clinic MGUS revised risk model includes serum FLCr 1.65, non-IgG MGUS and M protein >15 g/L.

All patients were diagnosed at the Amyloidosis and Myeloma Unit in the Department of Hematology (Hospital Clínic of Barcelona). Sample collection and clinical record review were performed after informed written consent in accordance with the Declaration of Helsinki. Study protocol was approved by the Institutional Review Board at Hospital Clínic of Barcelona. Patients were diagnosed according to standard International Myeloma Working Group criteria.

The “evolving” type was defined as a progressive increase of at least 10% in the M-protein size within the first 12 months from diagnosis when baseline M-protein was ≥30 g/L or over a period of 3 years (with a progressive increase in the M-protein size in each of the annual measurements) in patients with an initial M-protein <30 g/L (4, 11). Immunoparesis was defined as a decrease below normal levels of at least one of the uninvolved serum immunoglobulins. BM aspirates obtained at diagnosis were reviewed independently by two observers; plasma cell percentages were estimated from a 500-cell count by each examiner and the mean of the two values was considered for the analysis.

### RNA Isolation

CD138-depleted BM cell fraction was isolated with anti-CD138 mAb-coated immunomagnetic beads using an AutoMacs cell sorter (Milteny Biotec, Bergisch Gladbach, Germany). Purity was assessed after isolation, only samples with <2% of CD138+ cells were included in this study. Total RNA from the CD138^neg^ BM cell fraction was extracted using the TRIzol reagent (Thermo Fisher Scientific, Waltham, MA) according to manufacturer’s instructions.

### Gene Expression Analysis

RNA expression was measured with the nCounter technology; preparation and analyses were performed according to the manufacturer’s protocol (NanoString Technologies, Inc. Seattle, WA). A minimum of 100 ng of total RNA per sample was loaded and run on the HuV1_Cancer Immu_v1_1_Nanostring for analysis of the NanoString PanCancer Immune Profiling Panel of 730 immune-associated genes and 40 housekeeping genes. Expression counts were then normalized using the nSolver 4.0 software and custom scripts in R 3.6.3. Unpaired significance analysis of microarrays (SAM), using False Discovery Rate [FDR], were used to identify differential gene expression across sample groups. In addition, we calculated scores for immune-related gene expression signatures according to previously published literature, the “Tumor Inflammation Signature” (TIS) reported by Ayers et al. (12) and a previously described “cytolytic score” for hematological malignancies (**Supplementary Table S1**). The gene expression data have been deposited in the Gene Expression Omnibus database (http://www.ncbi.nlm.nih.gov/geo/) under accession number GEO: GSE186537.

### Statistical Analysis

Differences in time to progression (TTP) between patient groups were analyzed using Kaplan-Meier survival curves with the log-rank test used to indicate significance. Statistical differences for numerical values were calculated using the Brown–Forsythe ANOVA test, Mann-Whitney U test and the Kruskal-Wallis test. Spearman r was used to measure markers correlations. Differences were considered statistically significant at P values less than 0.05. All statistical analyses were performed using GraphPad Prism, v8.0.1 (GraphPad Software, Inc. San Diego, CA).

## Results

### Upregulation of Gene Sets Associated With Cytotoxicity and T Cell Functions in Patients With SMM Compared to MGUS

**Figure 1:**
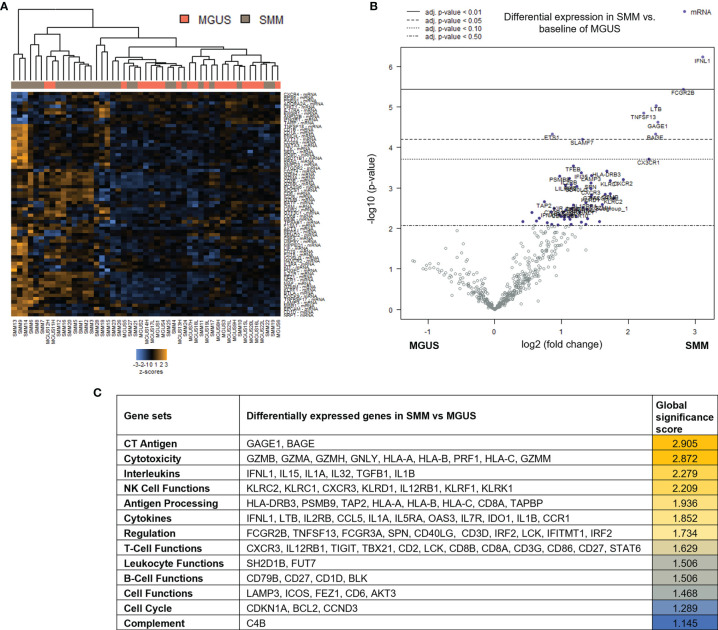
Genes associated with cytotoxicity were significantly upregulated in patients with SMM compared to MGUS. **(A)** Heatmap showing the unsupervised hierarchical clustering of patients with MGUS (n=22) and with SMM (n=28) based on the NanoString PanCancer Immune panel. **(B)** Volcano plot showing differentially expressed genes in SMM compared to MGUS. **(C)** Ranking of gene set functions according to the Global Significance Score quantified by NanoString software nSolver v.4.0.

Patients with SMM have a higher number of BMPC than patients with MGUS, which is associated to a higher risk of myeloma progression. In order to evaluate changes in the immune microenvironment associated to the increase in progression risk, we first performed gene expression analysis of CD138-depleted BM cells of 28 patients with SMM compared to 22 patients with MGUS. Most of the patients with SMM showed a distinct gene profile by unsupervised hierarchical clustering (**Figure 1A**). Among the 127 genes differentially expressed in SMM, only 4 genes were downregulated (*FLT3, ARG1, FCER1A* and *S100A12*) (**Figure 1B** and **Supplementary Table 2**). The top upregulated genes in SMM included molecules associated to myeloma (*SLAMF7* (CS1), *TNFSF13* (APRIL)) key molecules in cytotoxicity (*GZMB, GZMA, GZMH, GNLY, HLA-A, HLA-B, PRF1, HLA-C, GZMM*) and interleukins playing crucial roles in T cell functions (*IFNL1, IL15, IL1A, IL32, TGFB1, IL1B*) (**Figures 1B, C**). Although our results showed an upregulation of genes associated to cytotoxicity in the BM of patients with SMM compared to MGUS, we also found overexpression of inhibitory molecules such as LAG-3, TIGIT and IDO1, which may affect the anti-myeloma immune response in SMM.

### Genes Associated With NK and T Cell Functions Were Differentially Expressed in Patients With SMM Compared to Symptomatic MM

**Figure 2:**
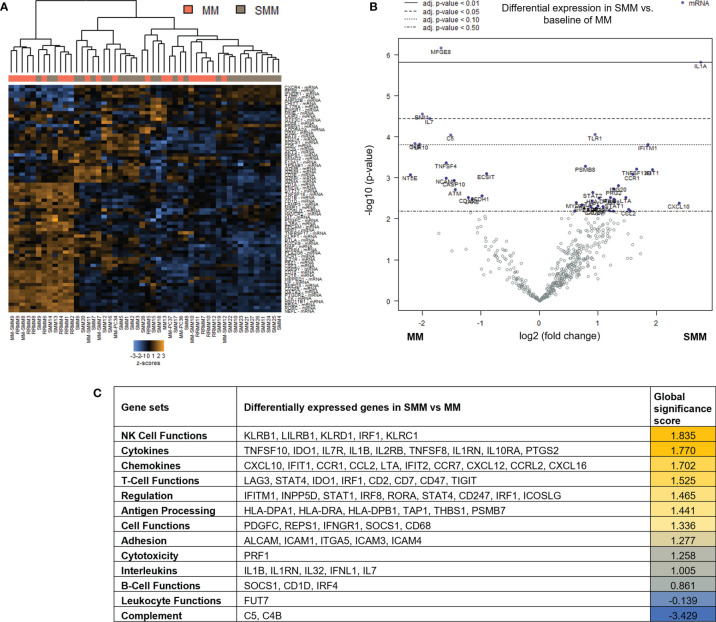
Genes associated with NK and T cell functions were differentially expressed in patients with SMM compared to symptomatic MM **(A)** Heatmap showing the unsupervised hierarchical clustering of patients with MM (n=22) and with SMM (n=28) based on the NanoString PanCancer Immune panel. **(B)** Volcano plot showing differentially expressed genes in SMM compared to MM. **(C)** Ranking of gene set functions according to the Global Significance Score quantified by NanoString software nSolver v.4.0.

To assess changes in immune microenvironment of patients with SMM compared to symptomatic MM, we analyzed gene expression data from the 28 patients with SMM compared to 22 patients with MM. Unsupervised hierarchical clustering did not show a distinct gene profiling discriminating patients with SMM *versus* MM underlying the wide heterogeneity found in SMM (**Figure 2A**). Among the 136 genes differentially expressed in SMM, 30 genes were downregulated and 106 upregulated in SMM compared to MM (**Figure 2B** and **Supplementary Table 2**). Global significance score showed that gene sets associated to NK functions were overrepresented in SMM (*KLRB1, LILRB1, KLRD1, IRF1, KLRC1*) (**Figure 2C**). Upregulation of inhibitory receptors such as KLRB1 (CD161), LILRB1 (CD85j), and KLRD1 (CD94) may indicate impairment in NK cell cytotoxicity. Accordingly, we also observed an increase in inhibitory checkpoints that can be expressed in both NK and T cells such as LAG-3 and suppressors of anti-tumor immunity such as IDO1 (**Figure 2C** and **Supplementary Table 2**). In patients with symptomatic MM, we found higher levels of genes previously associated with MM (*NCAM1, ATM, CD163, IL32*) and genes highly expressed in regulatory T cells (Tregs) such as *MFGE8, NT5E* (CD73) and *TIGIT*.

Out of the 10 patients with SMM which progressed to MM during follow up, 5 were available for gene expression analysis after progression. Paired gene expression analysis before and after progression showed a lower number of differentially expressed genes. Interestingly, the 54 upregulated genes in the SMM stage included several members of the TNF family (*TNFRSF9* (CD137), *TNFSF8, TNFRSF14, TNFRSF1A*) but also the ITIM-bearing inhibitory receptors PVR and LILRB3 (**Supplementary Figure S1**).

Taken together, our results could be indicative of an enrichment in gene sets associated to cytotoxic immune response in patients with SMM. However, the upregulation of inhibitory receptors may also suggest an exhausted phenotype in the cytotoxic cell compartment.

### Highly Expressed Genes Associated With Cytotoxic T Cell Function Correlated With Transcription Factors Tbet and Eomes in SMM

**Figure 3:**
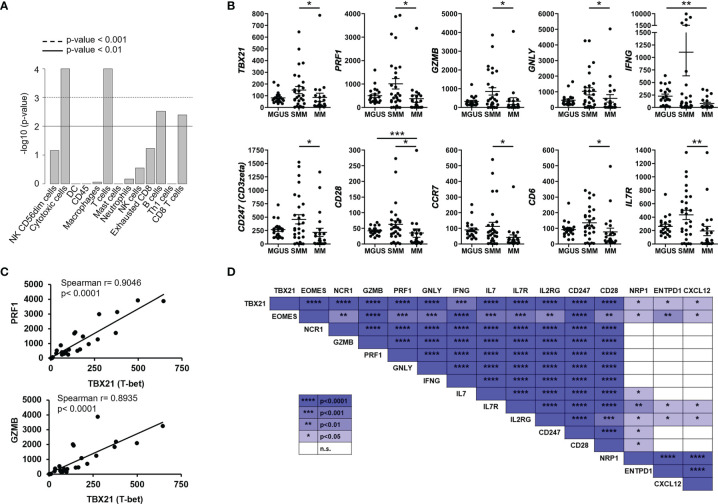
Highly expressed genes associated with cytotoxic T cell function correlated with transcription factors Tbet and Eomes in SMM. **(A)** Statistical analysis of cell types involved in SMM compared to MM. **(B)** Gene expression of genes significantly upregulated in patients with SMM. Kruskal Wallis test *p<0.05, **p<0.01, ***p<0.001. **(C)** Positive correlation between transcription factor Tbet (*TBX21*) and both perforin (*PRF1*) and granzyme b (*GZMB*). Spearman r and p values are indicated. **(D)** Summary of correlation analyses in gene expression in patients with SMM.

When comparing the tumor microenvironment in SMM with MGUS and MM, we found that in both cases the most relevant cell type was cytotoxic CD8^+^ T cells (**Figure 3A**). Indeed, key molecules in cytotoxicity such as Tbet (*TBX21*), perforin (*PRF1)*, granzyme b *(GZMB)* and granulysin (*GNLY*) were significantly increased in SMM compared to MM (**Figure 3B**). Although these molecules are also important in NK cytotoxicity, we observed an increase in molecules playing crucial roles in T cell functions such as CD3zeta (*CD247*), co-stimulatory receptor CD28 and CD6, consistent with the relevance of cytotoxic CD8^+^ T cells in SMM. Furthermore, expression of transcription factors Tbet and Eomes strongly correlate with key molecules in cytotoxicity such as perforin (*PRF1)*, granzyme b *(GZMB)* and granulysin (*GNLY*) in patients with SMM (**Figures 3C, D**). Both transcription factors also correlate with genes important for T cell function such as *IFNG, IL7, IL7R, IL2RG, CD247* and *CD28*. Of note, the natural cytotoxicity triggering receptor 1 *NCR1* (also known as NKp46, LY94) also correlated with these key genes suggesting that NK cells could also play a role in anti-myeloma cytotoxicity in SMM. As expected, cytotoxic molecules did not correlate with genes involved in Treg function such as *NRP1, ENTPD1*(CD39) and *CXCL12* (**Figure 3D**). As shown in **Figure 3B**, patients with SMM showed a wide range of expression levels, suggesting the presence of subgroups with differences in the composition and activation levels of the immune cells in the BM microenvironment.

### Gene Profiling of Bone Marrow Cells Identified Distinct Clusters in Patients With SMM Based on Immune Cell Composition and Activation Markers

**Figure 4:**
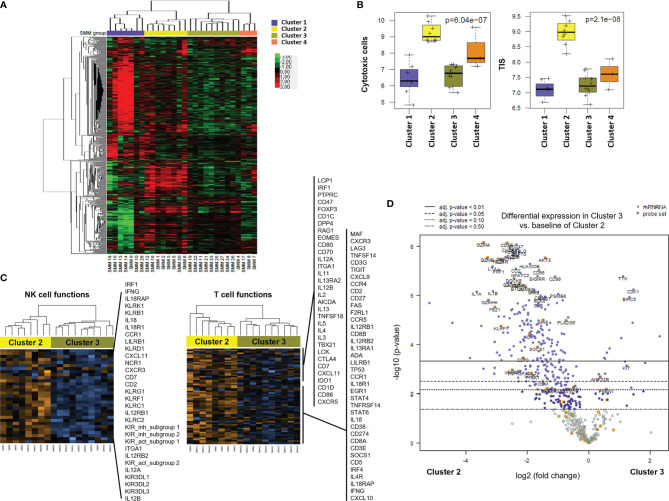
Gene profiling of bone marrow cells identified distinct clusters in patients with SMM based on immune cell composition and activation markers. **(A)** Heatmap showing the unsupervised hierarchical clustering of patients with SMM (n=28) based on the NanoString PanCancer Immune panel. **(B)** Statistical analysis of gene set associated to cytotoxic cells and the tumor inflammation signature (TIS) in the 4 distinct clusters of patients with SMM. **(C)** Heatmaps of genes associated to NK and T cell functions comparing cluster 2 *versus* 3. **(D)** Volcano plot showing genes differentially expressed in cluster 2 *versus* cluster 3.

Unsupervised hierarchical clustering after gene expression analysis with the PanCancer Immune panel classified the 28 SMM patients in four clusters (**Figure 4A**). Among the top upregulated genes in cluster 1 (n=7) we found cytokines and chemokines (*CCL11, CCL13, CCL16, CCL17, CCL21, CCL24, CXCL13, CX3CL1, XCR1, IFNL1, IFNL2, IL13, IL17A, IL25, LTB, TGFB2, TNFSF13*) and cytokine receptors (*TNFRSF12A, IL17RB*). In contrast, cluster 2 (n=8) showed an expression profile notably enriched for T and NK cell markers and signaling (*PRF1, GZMA/B/H/K, GNLY, CD2, CD3D/E, KLRD1, KLRF1, KLRB1, KLRK1, CD274 (*PD-L1*), LAG3*) and interferon signaling (*IFNG, MX1, ISG15, STAT1/2/4*) (**Figure 4A** and **Supplementary Table 2**). Indeed, the analysis of the expression of gene expression signatures (GES) associated with specific immune cell-types showed that cluster 2 included a distinct immune signature characterized by higher levels of transcripts associated to cytotoxic cells (**Figure 4B**). We also evaluated the tumor inflammation signature (TIS), which contains IFN-γ responsive genes associated with T cell activation, which has been shown to predict response to Programmed Cell Death Protein 1 (PD-1) blockade across multiple solid tumors (13). The TIS score correlated with the CD8^+^ T cell and cytotoxic cell signatures and was significantly higher in patients from cluster 2 (**Figure 4B**). Furthermore, gene expression analysis of cluster 2 compared to cluster 3 (n=10) showed a significant increase in genes associated to NK and T cell function in cluster 2 (**Figure 4C** and **Supplementary Table 2**). Indeed, most of the genes upregulated in SMM compared to MGUS were upregulated in cluster 2 compared to cluster 3 suggesting that the differences between SMM and MGUS were due to cluster 2 of patients with SMM while cluster 3 of SMM patients were more similar to a MGUS immune profile (**Figure 4D**). Therefore, although patients from cluster 2 showed an immune signature associated to a strong cytotoxic response, they also upregulated the expression of inhibitory checkpoints (*CD96, LAG3, BTLA, KLRB1*) which raised the question of whether this immune signature has an impact in progression to myeloma.

### Patients With SMM in Cluster 3 With Lower Expression of Cytotoxic Associated Molecules Showed Significantly Longer Progression Free Survival

**Figure 5:**
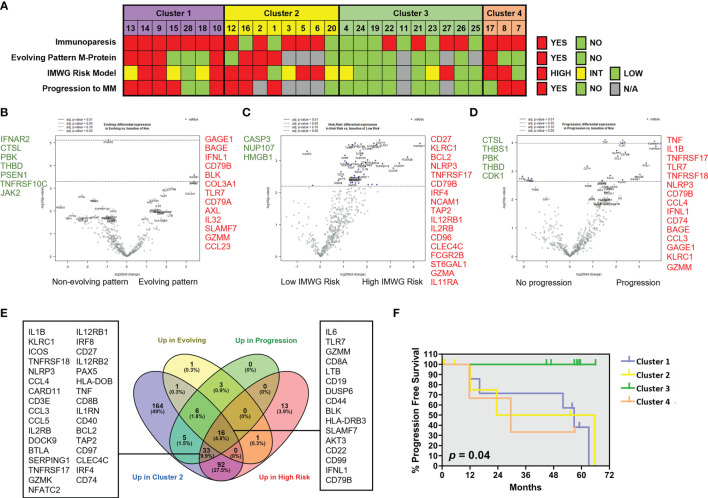
Patients with SMM with cytotoxic immune signature showed high-risk characteristics. **(A)** Clinical characteristics in patients with SMM divided into 4 clusters according to the results of the unsupervised hierarchical clustering using the PanCancer immune panel. Immunoparesis was defined qualitatively as one or more of uninvolved immunoglobulins below the normal levels. The International Myeloma Working Group (IMWG) SMM revised risk model includes serum M-protein >2 g/dL, involved to uninvolved free light-chain ratio >20 and BMPC infiltration >20%. *Patients enrolled in clinical trials were unavailable for determination of the M-protein behavior or progression to symptomatic disease. **(B)** Volcano plot showing genes differentially expressed in patients with evolving pattern of M-protein. **(C)** Volcano plot showing genes differentially expressed in patients with high progression risk according to IMWG. **(D)** Volcano plot showing genes differentially expressed in patients that progress to asymptomatic MM. **(E)** Venn diagram to assess common upregulated genes in cluster 2 and in patients with higher risk of progression. **(F)** Kaplan-Maier plot showing progression free survival (PFS) of patients in 4 clusters. Long-rank test.

We next analyzed the association between clinical parameters used to assess risk to progression in patients with SMM and their gene immune signatures (**Figure 5A**). SMM patients with an evolving pattern of the M-protein showed an increase in genes associated to myeloma (*SLAMF7, CD79A, CD79B*) and AXL (**Figure 5B** and **Supplementary Table 2**). Some of these molecules are expressed in a variety of immune cell types but also may be found in PCs, thus we cannot rule out that this high-sensitivity gene expression analysis may detect presence of remaining PCs. However, our experimental approach can still capture major differences in key immune genes of interest. In high-risk SMM patients, 155 upregulated genes included molecules associated to MM (*TNFRSF17*, *NCAM1*, *IRF4, CD79B*), cytotoxic molecules (*KLRC1, GZMA*) and AXL (**Figure 5C**). The median follow up of the patients with SMM was 5.3 years. Ten patients with SMM progressed to symptomatic MM, with a median time to progression (TTP) of 1.9 years. SMM patients that progressed to MM showed 76 differentially expressed genes when compared to SMM patients who showed no progression during follow up, with an increase in 63 including molecules involved in immune response activation such as TNF, IL-1B and granzyme M (**Figure 5D**). Most of the genes upregulated in high-risk patients were included in the list of upregulated genes in cluster 2 (**Figure 5E**). We next wanted to assess whether distinct clusters based in immune signatures were associated to differences in progression free survival (PFS). Four patients in cluster 2 were excluded from this analysis since they started treatment in a clinical trial for high-risk SMM patients (GEM-CESAR). As shown in **Figure 5F**, log-rank test showed that patients in cluster 3 with lower expression of cytotoxic genes and an immune signature more similar to MGUS patients had a significantly longer PFS compared to the other of clusters (*p*=0.04).

Some of the studied genes were independently associated with shorter PFS such as *TNF, TNFAIP3, GZMM* and *TNFRSF14* (also known as HVEM) (**Supplementary Figure 2A**). Conversely, high expression of transcripts for MAPK14, LTF, SMAD3, FOS, PSEN1 and THBS1 were associated with increased progression free survival (**Supplementary Figure 2B**). Taken together, our results could indicate that genes associated to an exhausted phenotype in cytotoxic T cells are upregulated in high-risk SMM patients while some members of the TNF superfamily are significantly associated to myeloma progression.

## Discussion

One of the most important challenges in the fight against MM is the assessment of progression risk in patients with asymptomatic conditions that precede this malignancy. Several algorithms have been developed based on clinical and laboratory parameters to identify high-risk patients (5, 6). To better understand the basic mechanisms of myeloma progression in patients with SMM, most of the genetic studies have focused on investigating CD138^+^ PC characteristics such as chromosomal aberrations (14), gene expression profiling (15), whole-exome sequencing of clonal PC (16–18). However, PC extrinsic factors in the BM microenvironment may also play a crucial role in myeloma progression (8). In this regard, several studies have reported impairment of immune cell functions in symptomatic MM compared to MGUS (19, 20). In addition, immune cells from peripheral blood of high-risk SMM patients have shown an impaired immune system that could be reactivated by therapeutic immunomodulation to delay the progression to MM (21). Here, we wanted to investigate the immune cell compartment in the BM of patients with SMM to dissect the immune evasion strategies involved in malignant PC survival, with important clinical implications for patient risk stratification and early treatment.

In this study, we investigated changes in the BM immune microenvironment in SMM that could affect the efficacy of the immune response against malignant PC and, consequently, the time to symptomatic MM progression. Our results showed an upregulation of genes associated to cytotoxicity in SMM compared to both MGUS and symptomatic MM. However, we also found overexpression of inhibitory molecules such as LAG-3, TIGIT and IDO1, which may affect the anti-myeloma immune response in SMM. Patients with SMM showed a wide heterogeneity in their immune compartment (22), which let us to identify 4 clusters based on their gene expression profiles. Importantly, patients in cluster 2 had higher expression of cytotoxic molecules (GZMB, PRF, GNLY, IFNG) but also upregulation of some inhibitory molecules (LAG3, KLRC1, CD96, BTLA), suggesting the presence of exhausted T cells. Consistently, features of exhaustion in both CD4^+^ and CD8^+^ T cells, such as a significant increase in PD-1 and CTLA-4 compared to healthy donors, have been reported in the BM of patients with symptomatic MM (23). Interestingly, a recent analysis based on single-cell RNA sequencing of BM cells from 8 patients with SMM compared to 4 healthy controls also showed an increase in negative immune checkpoints such as LAG-3 and TIGIT (24). Our study, including a larger cohort of patients with SMM and comparing them with asymptomatic MGUS and active MM, revealed distinct clusters in SMM based on their immune signature associated to risk of progression. However, not all inhibitory immune checkpoints were overexpressed in SMM. For instance, PD-1 was not significantly expressed in SMM microenvironment, suggesting that PD-1 may not play a crucial role in myeloma progression. This finding would be consistent with the negative results obtained in clinical trials blocking PD-1 signaling in MM (25) and in SMM (26). On the contrary, patients with SMM in cluster 2 showed a significant increase in the ITIM-bearing inhibitory receptor TIGIT (27, 28) which is consistent with our previous findings demonstrating that TIGIT blockade can be a useful therapeutic strategy in patients with SMM and active MM with Nectin-2 expressing PC (29). Thus, the role of TIGIT-Nectin-2 interaction in myeloma progression and its relevance as therapeutic target in patients with SMM remains to be elucidated.

Finally, paired gene expression analysis showed several members of the TNF family (*TNFRSF9* (CD137), *TNFSF8, TNFRSF14 (*HVEM)*, TNFRSF1A*) upregulated genes in the SMM stage compared to active MM. In this regard, we also found an association of high expression of TNF superfamily members (*TNF, TNFAIP3*, *TNFRSF14 (*HVEM)) with a significantly shorter PFS in patients with SMM suggesting that the presence of a pro-inflammatory microenvironment may contribute to myeloma progression instead of generating an efficient anti-myeloma response. Thus, chronic inflammation and exhaustion of cytotoxic T cells may partly explain a defective control of malignant PC growth in SMM. Our findings may contribute to a better understanding of the immune dysfunction mechanisms underlying multiple myeloma progression (30). Since there is no specific treatment for patients with SMM, high-risk patients are treated with agents approved for symptomatic MM. In MM, several immunotherapies have been approved against CD38 (31), signaling lymphocytic activation molecule F7 (SLAMF7) and B cell maturation antigen (BCMA) (32, 33). In this study, we found several inhibitory immune checkpoints (TIGIT, CD96, BTLA, LAG3, KLRC1) upregulated in high-risk SMM patients, some of them being currently tested in clinical trials for patients with relapsed refractory MM, such as dual blockade of TIGIT and LAG-3 (NCT04150965). Further research is needed to evaluate the relevance of these molecules as potential therapeutic targets to avoid myeloma progression in SMM.

## Conclusions

In summary, our results provide insight into the composition and activation levels of the BM immune cells from distinct clusters of patients with SMM which may have an impact in progression to symptomatic disease. Gene expression profiling of BM cells surrounding malignant PC revealed changes in genes associated to exhausted cytotoxic T cells that can be relevant as biomarkers to better characterize the progression risk of asymptomatic patients with SMM. Furthermore, our findings could be useful to guide the implementation of current approved treatments and to develop new targeted immunotherapies.

## Data Availability Statement

The datasets presented in this study can be found in online repositories. The names of the repository/repositories and accession number(s) can be found below: GEO, GSE186537.

## Ethics Statement

The studies involving human participants were reviewed and approved by Institutional Review Board at Hospital Clínic of Barcelona (HCB/2019/0382). The patients/participants provided their written informed consent to participate in this study.

## Author Contributions

II performed experiments, analyzed data and wrote the manuscript. FB-M analyzed gene expression results. DM, AO-C, LR-L, MC, JB, and LR recruited patients and collect clinical data. M-PM, BM-A, LP, and EL performed experiments and data analysis. AP provided reagents and helped with Nanostring technology. EL and CF designed research, analyzed data and wrote the manuscript. All authors contributed to the article and approved the submitted version.

## Funding

This work was supported in part by Grants PI16/00423, PI19/00669 and PI20/00436 from Instituto de Salud Carlos III (Ministerio de Economía y Competitividad, co-funded by Fondo Europeo de Desarrollo Regional (FEDER)-Una manera de Hacer Europa) and the CERCA Programme/Generalitat de Catalunya.

## Conflict of Interest

The authors declare that the research was conducted in the absence of any commercial or financial relationships that could be construed as a potential conflict of interest.

## Publisher’s Note

All claims expressed in this article are solely those of the authors and do not necessarily represent those of their affiliated organizations, or those of the publisher, the editors and the reviewers. Any product that may be evaluated in this article, or claim that may be made by its manufacturer, is not guaranteed or endorsed by the publisher.

## Supplementary Material

Supplementary Figure 1 Genes upregulated in 5 patients with SMM compared to their gene profile after progression to symptomatic MM **(A)** Heatmap showing the unsupervised hierarchical clustering of 5 patients before and after progression. **(B)** Expression of ITIM-bearing receptors, poliovirus receptor (PVR) and leukocyte immunoglobulin like receptor B3 (LILRB3) before and after progression.

Supplementary Figure 2 Analysis of progression free survival *versus* genes differentially expressed in patients with SMM. **(A)** Kaplan-Maier plot showing progression free survival (PFS) of patients (n=28) *versus* indicated genes. Upregulation of TNF superfamily members were associated to shorter PFS. Long-rank test *p* values are indicated. **(B)** High expression of genes such as MAPK14, Thrombospondin 1 (*THBS1*), transcription factor FOS and SMAD3 were associated to longer PFS. Long-rank test *p* values are indicated.

## Abbreviations

BM, Bone marrow; ISS, International stage system; MGUS, Monoclonal gammopathies of undetermined significance; MM, Multiple myeloma; NK, Natural killer; PC, Plasma cells; SMM, Smoldering MM; Th, T helper.
