# Supplementary figures and images for: Gene Expression Analysis of the Bone Marrow Microenvironment Reveals Distinct Immunotypes in Smoldering Multiple Myeloma Associated to Progression to Symptomatic Disease
Source: Front Immunol. 2021 Nov 22;12:792609. doi: 10.3389/fimmu.2021.792609 (PMC8646031; doi:10.3389/fimmu.2021.792609)

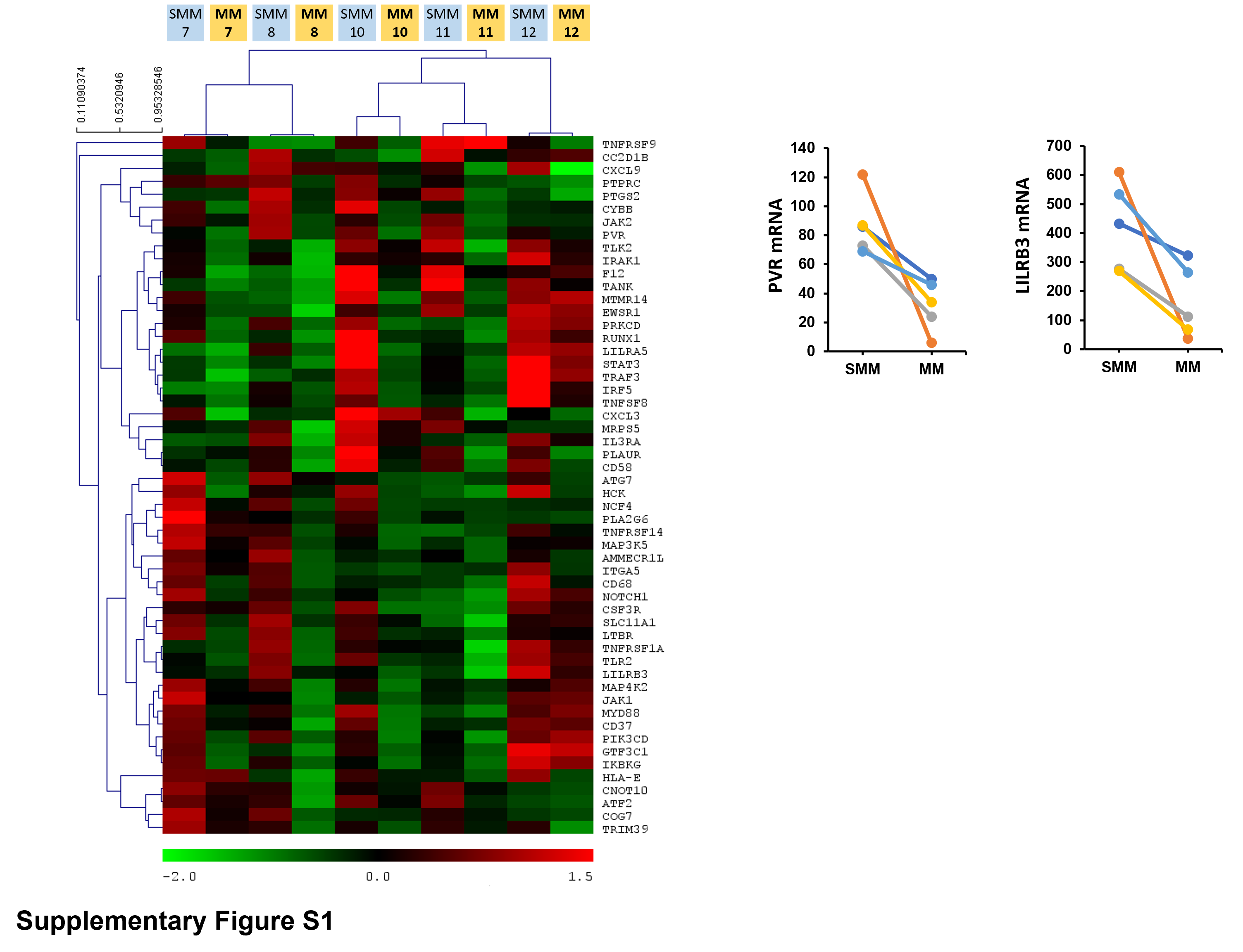

Supplement: Supplementary Figure 1 — Genes upregulated in 5 patients with SMM compared to their gene profile after progression to symptomatic MM (A) Heatmap showing the unsupervised hierarchical clustering of 5 patients before and after progression. (B) Expression of ITIM-bearing receptors, poliovirus receptor (PVR) and leukocyte immunoglobulin like receptor B3 (LILRB3) before and after progression. [file Image_1.tif]

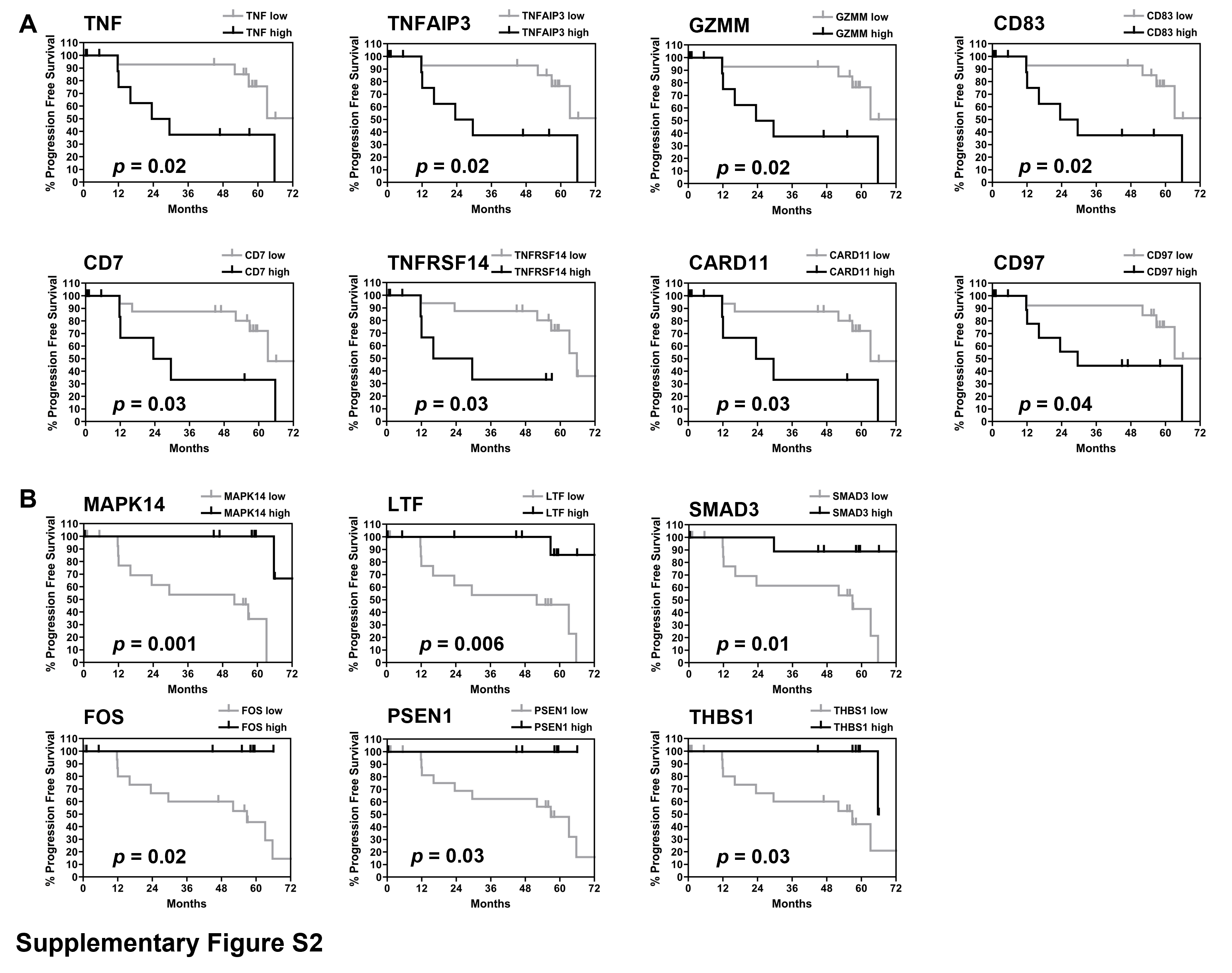

Supplement: Supplementary Figure 2 — Analysis of progression free survival versus genes differentially expressed in patients with SMM. (A) Kaplan-Maier plot showing progression free survival (PFS) of patients (n=28) versus indicated genes. Upregulation of TNF superfamily members were associated to shorter PFS. Long-rank test p values are indicated. (B) High expression of genes such as MAPK14, Thrombospondin 1 (THBS1), transcription factor FOS and SMAD3 were associated to longer PFS. Long-rank test p values are indicated. [file Image_2.tif]
